# The Hebb repetition effect in complex span tasks: Evidence for a shared learning mechanism with simple span tasks

**DOI:** 10.3758/s13421-021-01261-3

**Published:** 2021-12-06

**Authors:** Claudia Araya, Klaus Oberauer, Satoru Saito

**Affiliations:** 1 Department of Cognitive Psychology, Graduate School of Education, Kyoto University, Yoshida-honmachi, Sakyo-ku, Kyoto, 606-8501 Japan; 2 Japan Society for the Promotion of Science, Tokyo, Japan; 3 Department of Psychology, University of Zurich, Zürich, Switzerland

**Keywords:** Hebb repetition learning, Working memory, Long-term memory, Complex span tasks, Simple span tasks

## Abstract

The Hebb repetition effect on serial-recall task refers to the improvement in the accuracy of recall of a repeated list (e.g., repeated in every 3 trials) over random non-repeated lists. Previous research has shown that both temporal position and neighboring items need to be the same on each repetition list for the Hebb repetition effect to occur, suggesting chunking as one of its underlying mechanisms. Accordingly, one can expect absence of the Hebb repetition effect in a complex span task, given that the sequence is interrupted by distractors. Nevertheless, one study by Oberauer, Jones, and Lewandowsky (2015, *Memory & Cognition*, *43*[6], 852–865) showed evidence of the Hebb repetition effect in a complex span task. Throughout four experiments, we confirmed the Hebb repetition effect in complex span tasks, even when we included distractors in both encoding and recall phases to avoid any resemblance to a simple span task and minimized the possibility of chunking. Results showed that the Hebb repetition effect was not affected by the distractors during encoding and recall. A transfer cycle analysis showed that the long-term knowledge acquired in the complex span task can be transferred to a simple span task. These findings provide the first insights on the mechanism behind the Hebb repetition effect in complex span tasks; it is at least partially based on the same mechanism that improves recall performance by repetition in simple span tasks.

In 1961, Donald Hebb developed an experiment in which participants were required to remember a sequence of numbers from 1 to 9 presented in random order for every trial, except for every third trial, where the same series of numbers was repeated, without informing the participants. Hebb (1961) found that the repetition led to improvement of the immediate serial recall in comparison with the random lists. The improvement of recall on a repeated list (e.g., every three trials) over nonrepeated lists is called the Hebb repetition effect (Couture et al., 2008; Cumming et al., 2003; Page & Norris, 2009). Hebb repetition learning, which is the basis for the effect, has been shown to be closely related to language acquisition and vocabulary learning (Mosse & Jarrold, 2008; Page & Norris, 2009), and is even considered a laboratory analogue of naturalistic word-form acquisition (Mosse & Jarrold, 2008; Page & Norris, 2008, 2009; Szmalec et al., 2009; Szmalec et al., 2012). It is an example of long-term sequence learning, and relies on the cognitive processes responsible for representing serial order information in memory, as is phonological word-form learning (Szmalec et al., 2012). Experiments using nonwords have demonstrated that repeated sequences learned by Hebb repetition learning establish novel phonological word-forms in lexical memory (Mosse & Jarrold, 2008; Szmalec et al., 2009; Szmalec et al., 2012). Therefore, understanding the mechanisms behind the Hebb repetition effect can give us important insight on how sequence knowledge is acquired and how to improve it.

The Hebb repetition effect has been demonstrated several times using standard immediate serial recall tasks (i.e., simple span tasks; Page et al., 2006; Page et al., 2013; Smalle et al., 2016; St-Louis et al., 2019). It seems that what we learn through Hebb repetition is mainly the serial order of the items in the repeated list (Hitch et al., 2005), but its underlying mechanism is still unclear.

Previous research has shown that both the relation of items to their list position and their relation to neighboring items need to be the same on each repetition list for the Hebb repetition effect to occur. On the one hand, Hebb repetition learning could only be found when memory items were repeatedly presented at the same serial positions from the beginning. When the repeated sequences were presented starting from different serial positions, the learning effect was small (Hitch et al., 2005). On the other hand, when only the odd or even positions are repeated, the learning effect was observed only at the first few repeated positions (Hitch et al., 2005). In this situation, although half of the memory items were repeatedly presented at the same serial positions, their neighboring items changed over the repetition. Furthermore, learning of a whole repeated list has not been found to transfer to a list were only the odd, or only the even positions are repeated (Cumming et al., 2003). These results have led to the conclusions that (a) repetition of the sequence as a whole from the start of the list is necessary for the occurrence of the effect, (b) that learning of item-to-item associations alone cannot explain the Hebb repetition effect, and (c) that position–item associations alone cannot explain the Hebb repetition effect.

One possible mechanism for the repetition effect could be chunking, that is, the effect emerges from creating a unified long-term representation of the input sequence. Two computational models of the Hebb repetition effect (Burgess & Hitch, 2006; Page & Norris, 2009) use different variants of chunk representations in long-term memory to explain it. What they have in common is that they represent each list in long-term memory separately, and as a unit that is retrieved in an all-or-none manner: If, and only if, a new input sequence matches the long-term memory representation sufficiently, it is retrieved as a whole, and contributes to recall.

Complex span tasks refer to a variant of serial recall in which, unlike simple span tasks, stimuli that do not need to be remembered (i.e., distractors) are interspersed between each memory item (Daneman & Carpenter, 1980). Given the previous findings, in complex span tasks one can expect the absence of the Hebb repetition effect (Oberauer et al., 2015) for the following reason: Distractors, like memory items, are encoded into working memory (Oberauer et al., 2012a, b; Oberauer & Lewandowsky, 2016). Therefore, when Hebb lists are repeated in a complex span paradigm, while the distractors in between items are always novel, the relation between neighboring list items are interrupted by the intervening distractors, so it is difficult to learn associations between neighboring items. Likewise, learning the memory list through chunking becomes challenging because a chunk that unifies the entire input sequence—including the distractors—would not match a repeated presentation of the same list, interleaved with different distractors.

However, Oberauer et al. (2015) showed evidence of the Hebb repetition effect in a complex span task. They initially suggested that one possibility was that the participants can learn the sequence during the recall phase. In their second experiment, they moved the distractors to the recall phase, and again, the Hebb repetition effect was observed. Yet, in both cases, some simple-span aspects were present—that is, there was an uninterrupted sequence either during encoding or during recall. Therefore, either at encoding or at recall, both relations deemed necessary for Hebb repetition learning (i.e., relations of items to positions, and relations between neighboring items) remain constant, giving participants a chance to create integrated representations, perhaps contributing to the Hebb repetition effect. To conclude, the experiments of Oberauer et al. (2015) gave the first insights on the topic, but these were not enough to confirm that the Hebb repetition effect can occur when the items are never presented and recalled in immediate succession.

In a series of four experiments, we first establish the presence of the Hebb repetition effect in complex span tasks. Experiment 1 was a replication of Oberauer et al.’s (2015) second experiment, and the results were very similar to the original, demonstrating that the Hebb repetition effect is present in a complex span task. However, there are still several variables that could be playing an important role in the appearance of this effect. Experiments 2 and 3 had two main objectives: One was to investigate whether the Hebb repetition effect occurs when memory-list items are never experienced in immediate succession, neither during the encoding nor recall phase, by including distractor tasks in both phases. The other objective was to examine whether Hebb repetition learning during the complex span task can be transferred to a simple span task. The results of Experiments 2 and 3 showed both Hebb repetition and transfer effects. Experiment 4 had as an objective to extend the results of the preceding experiments to a task were the distractors and memory items were less distinctive, once again the Hebb repetition and transfer effects were found. The results found here question the conclusions from previous research regarding the mechanism underlying this effect, and call for a change of their theoretical understanding. Therefore, we propose modifications to the existing explanation for Hebb repetition learning.

## Experiment 1

The purpose of the first experiment was to replicate the results obtained by Oberauer et al. (2015).

### Method

#### Participants

Participants were 30 undergraduate and graduate students of Kyoto University who took part in a single 1-hour session in exchange for a 1000JPY book coupon. However, four participants had to be excluded from the analysis; three did not complete the task and one failed to follow instructions. Consequently, the total sample was of 26 participants (11 females and 15 males) with ages ranging from 18 to 33 years (*M* = 21.57). All participants were native Japanese speakers.

#### Materials

As in Oberauer et al. (2015), a list of all the consonants except *Q* and *Y* was used, and the memory list for each trial was created by randomly selecting a consonant without replacement until completing the desired quantity. The list for the first repeated trial (Trial 3) was constructed in the same way and then held constant for all repetitions (every third trial). The distractor task was a size judgment task: Participants had to decide whether a noun referred to an object larger or smaller than a soccer ball. We used the original experiment’s word list from Oberauer et al. (2015). The objects in the list varied across a broad range of size, from “ladybird” to “sun,” but only the 25% smallest and 25% largest objects were chosen to avoid ambiguity. Therefore, as in Oberauer et al. (2015), 264 nouns referring to concrete objects were selected and translated to Japanese. Some words from the original list had to be replaced due to difficulties in the translation. The words were selected at random on every trial including on the repetition trials.

#### Procedure

The same procedure as in Oberauer et al. (2015) was employed. The task consisted in a total of 24 trials. Each trial started with a fixation cross that lasted 3 s, followed by the first consonant (memory item) displayed centered and in red for 1.5 s. The letter was immediately replaced by the first distractor word, displayed in black, until the participants gave a response, or for a maximum of 2 s. Participants were instructed to make a size judgment deciding whether the object was smaller or larger than a soccer ball, and were required to respond by pressing the corresponding key on the computer keyboard—the left arrow key if the object was smaller or the right arrow key if the object was larger. Each trial consisted of eight consonants; each letter was followed by four size judgment tasks, and so on. After the last word, a red question mark was shown, prompting the participants to recall the memory items in the same order as presented by pressing the letters on the computer keyboard. The entered letter was displayed for 0.3s, followed by a red question mark and so on for the eight letters on the memory list. Omissions were not allowed. The next trial began 2.5 s after the last recall response. Between each trial, a screen asking the participants to press the space bar to continue was added, giving them the opportunity to take a break if necessary. However, the average response time for this key was 6.7 s, meaning that the participants did not take long breaks between trials.

### Results

The data were analyzed with a Bayesian linear regression model, using the *lmBF* function in the *Bayes Factor* package (Morey & Rouder, 2018; Rouder et al., 2012) for R (R Core Team, 2018). This function is used to estimate the Bayes factor (BF) of linear models, the BF reflects the relative strength of evidence for two models compared with each other (Dienes, 2014). Two given models can be compared indirectly by dividing their BFs from comparisons to the same reference model (usually a null model).

Two hypotheses were tested. First, whether the Hebb repetition effect is present; second, whether repetition of the memory list had an impact on speed and accuracy of the distractor task. For each analysis, the models included two predictors—cycle and repetition. Cycle refers to the ordinal number of the eight sets of three consecutive trial, including one repeated Hebb list and two nonrepeated filler lists; in total there were eight cycles per participant. Cycle was entered in the model as a continuous variable centered on its mean. Repetition refers to the comparison between the repeated list and the nonrepeated list. In total, there were eight Hebb lists and 16 filler lists. For each analysis, we estimated four models: M_*c*_, containing only the main effect of cycle; M_*r*_, with only the main effect of repetition; M_*add*_, with the additive effects of cycle and repetition; and M_*full*_, with both the additive effects and their interaction.

We evaluated the strength of evidence for the main effects by calculating the BF of each model relative to the more comprehensive model—that is, we estimated evidence for the interaction by BF(M_*full*_)/BF(M_*add*_) and chose the better model, then compared that model to a derived model in which the effect of interest was removed in order to assess the effect of each variable, that is, BF(M_*full*_)/BF(M_*full-cycle*_) and BF(M_*full*_)/BF(M_*full-repetition*_) or BF(M_*add*_)/BF(M_*add-cycle*_) and BF(M_*add*_)/BF(M_*add-repetition*_). BFs larger than 1 reflect evidence in favor of the model in the numerator, and BFs smaller than 1 reflect evidence in favor of the model in the denominator. The strength of evidence for the model in the denominator can be calculated by the reciprocal of the BF. For example, if BF(M_*full*_)/BF(M_*add*_) = 0.5, then the BF in favor of the additive model is 2. According to Kass and Raftery (1995) BF between 1 and 3 show evidence “barely worth mentioning”; between 3 and 10 show “substantial evidence”; between 10 and 100 show “strong evidence”; and >100 show “decisive evidence.”

#### Memory accuracy

**Fig. 1:**
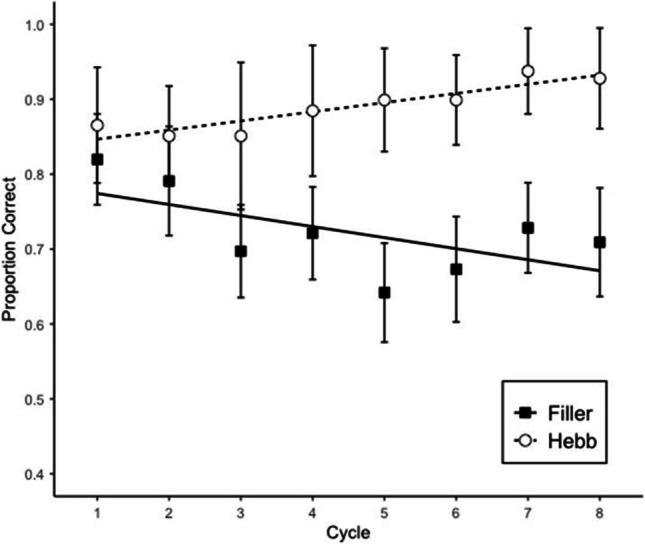
Memory accuracy in Experiment 1*.* Error bars are 95% confidence intervals (CIs) for within-subject comparisons (Bakeman & McArthur, 1996). The CIs can be interpreted in terms of classical null-hypothesis tests for pair-wise comparisons between data points: Two means differ significantly (p < .05) when their CIs overlap by less than 50% of the interval between each mean and the corresponding CI boundary (Cumming & Finch, 2005). The straight lines are regression lines estimated from fitting a linear model

Memory performance was scored as the proportion of letters recalled in their correct within-list position. Figure 1 shows proportion of correct answers by cycle and repetition (Filler vs. Hebb). Table 1 summarizes the BFs reflecting the strength of evidence for the main effects and the interaction. The analysis showed strong evidence for the interaction and for the main effect of repetition, indicating a strong contribution of the repetition variable to the main effect. There is no evidence for cycle—rather, we obtained substantial evidence for the model without the cycle variable. Effect sizes were estimated by sampling from the posterior distribution, using the *posterior* function in the *BayesFactor* package (Morey & Rouder, 2018). The results give information about the posterior mean and the 95% credible interval of the effect, which is the range in which the true effect size lies with a posterior probability of .95. In this case, the mean of the posterior effect of repetition was 0.16 with a 95% credible interval of 0.12–0.19. Based on these results we can say that the Hebb repetition effect increases memory performance in complex span by 12–19 percentage points over eight list repetitions. The results are similar to the ones of the previous experiment conducted by Oberauer et al. (2015).^1^

**Table 1:** Experiments 1–4: Bayes factors for the linear models

Effects	Experiment 1	Experiment 2	Experiment 3	Experiment 4
	*Memory accuracy*			
Cycle	0.15	0.20	4.21	174.5
Repetition	8.33 × 10^27^	5.65 × 10^4^	2.98 × 10^5^	56.4
Cycle × Repetition	54.4	0.53	0.17	6.68
	*Size-judgment accuracy*	*(Encoding/Recall)*		
Cycle	14.14	0.24/0.31	0.11/0.12	272.2/3860.6
Repetition	3387.7	12.09/17.91	5.59 × 10^2^/0.29	0.11/0.14
Cycle × Repetition	0.50	0.30/0.20	0.17/0.16	0.34/ .22
	*Size Judgment RT*	*(Encoding/Recall)*		
Cycle	2.07 × 10^9^	1.82 × 10^23^/5.93 × 10^8^	3.05 × 10^11^/6.95 × 10^6^	2.67 × 10^16^/2.73 × 10^24^
Repetition	3.19 × 10^7^	46.87/7.47 × 10^3^	4.40 × 10^7^/5.29 × 10^2^	0.24/1.13
Cycle × Repetition	0.23	0.20/0.52	0.29/0.14	0.10/0.11

#### Size judgment performance

**Fig. 2:**
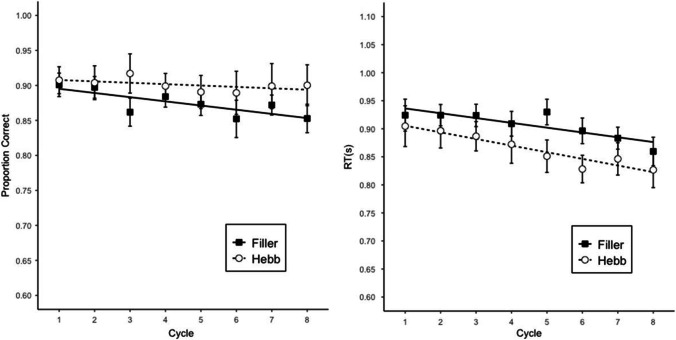
Performance in the size judgment task in Experiment 1. Error bars are 95% CIs for within-subject comparisons. The straight lines are regression lines estimated from fitting a linear model

Failures to respond to a size judgment trial within the allotted 2 s were scored as errors. We analyzed data including only response times (RTs) of correct responses. We estimated Bayesian linear models with the same predictors as for memory accuracy. The BFs are shown in Table 1, and the proportion of correct answers and RTs are presented in Fig. 2. There was no evidence for the interaction for either accuracy or RTs; therefore, the analysis was conducted with the additive model. There was strong evidence for the main effect of both cycle and repetition on accuracy and RTs. As in memory accuracy, the main effect of repetition shows that the list repetition had a beneficial effect on both accuracies and RTs.

### Discussion

In the present study, we successfully replicated the general patterns of the results of Experiment 2 in Oberauer et al. (2015), whose findings suggested that the Hebb repetition effect is present in a complex span task. We found a main effect of repetition and of the interaction between cycle and repetition on memory accuracy. The main effect of repetition is the key evidence for the Hebb repetition effect, as it can only come about by stronger learning in the Hebb than in the filler lists (Oberauer et al., 2015). Based on our findings, the Hebb repetition effect increases memory performance in complex span by 12–19 percentage points over eight list repetitions, a bit higher but similar to the improvement of 6–16 percentage points found by Oberauer et al. (2015). In both size judgment accuracy and RT, we found a main effect of cycle and repetition.

However, we found some differences in our results compared with the ones in Oberauer et al. (2015). First, there seems to be a decrease of memory performance in the filler lists (see Fig. 1), whereas previous studies normally show that the filler list performance remains stable throughout the task while the Hebb list increases in accuracy (Norris et al., 2018; Oberauer et al., 2015; Szmalec et al., 2009). This might be due to the proactive interference during the task. Given that the Japanese participants do not use as many alphabetical letters in daily life, the previously learned lists could be interfering with the current items more strongly in our participants than in those who use alphabetical languages. Another possibility is that as the task advances the participants become tired and their motivation decreases. Second, there was relatively small improvement in the memory performance of the Hebb lists. This is because the average memory accuracy was high already for the first Hebb trial, meaning that there was not much room for improvement throughout the task. Third, there was no main effect of cycle on memory accuracy. This could be caused precisely by the first two differences: the decrease of memory performance of filler lists over cycle and a relatively smaller improvement of the memory performance of the Hebb lists. Fourth, contrary to what was found in Oberauer et al.’s (2015) second experiment, we found strong evidence for the interaction of cycle and repetition of the memory accuracy. Oberauer et al. (2015) pointed out that the reason why they found a weak interaction effect could be that the repetition effect emerged early in the experiment. However, the same can be said for the results of the present experiment. We hypothesize that finding strong evidence for the interaction could also be due to the decrease of memory performance of the filler lists, creating a bigger gap between filler lists and Hebb list as the experiment moves forward, making the effect of cycle over the proportion of correct answers depend on the type of list. Finally, there was no interaction in the size judgment response time; a reason for this could be that the repetition effect can be seen since the earliest trials, meaning that the effect of repetition on response time does not depend on the cycle.

## Experiments 2 and 3

In the initial experiment of Oberauer et al. (2015) with a standard complex span task, the recall phase remained uninterrupted, leaving the possibility that Hebb repetition learning occurred at the time of recall. On that account, they developed a study in which they moved the size judgment task from the encoding to the recall phase, and still the results showed a clear Hebb repetition effect.

Whether the Hebb repetition effect occurs during encoding or recall has been an issue in the literature. Although some previous studies suggested that the Hebb repetition effect arises from learning of the output sequence (i.e., it occurs only during recall; Cohen & Johansson, 1967; Cunningham et al., 1984). A more recent study indicated that Hebb repetition learning occurs during both encoding and recall. Even though the effect is somewhat larger after recall, it can still be found when the repeated lists are only encoded (Oberauer & Meyer, 2009). Guerrette et al. (2018) investigated whether Hebb repetition learning of a repeated list transfers to a test of the repeated list with a new recall direction (forward vs. backward). They found that changing recall direction in the transfer test reduces but does not eliminate the advantage of the repeated list, consistent with the assumption that learning of both the presentation order and the recall order contributes to the Hebb repetition effect.

Therefore, we designed the following experiments with the aim of diminishing the possibility that participants are learning the sequence by forming integrated representations in either the encoding or the recall phase. In Experiments 2 and 3, we developed and applied a modified version of the first experiment task, by including distractors during the recall phase (i.e., distractors in both encoding and recall phase). Additionally, a cycle of three trials including two filler lists and one Hebb list without any distractors was included at the end of the task (i.e., the transfer cycle), with the aim of measuring if learning of the Hebb list in a complex span task can be transferred to a simple span task. Simply put, if there is a transfer effect then the evidence points towards the same mechanism in simple and complex span tasks; however, if the transfer effect cannot be found, then the mechanism might be different. Experiments 2 and 3 only differed in the cognitive load. Experiment 2 had a higher cognitive load with four distractors and 1.2 s to respond; in Experiment 3, the distractor task was modified to lower the cognitive load to ensure that the participants engage in the task properly. We increased the processing time back to 2 s (as in Experiment 1) and reduced the number of the distractors to two instead of four.

### Method

#### Participants

Participants were 30 (Experiment 2) and 30 (Experiment 3) undergraduate and graduate students of Kyoto University who took part in a single 1-hour session in exchange for a 1000JPY book coupon. However, two participants per experiment failed to complete the task and had to be excluded from the analysis. Consequently, the total sample for Experiment 2 was of 28 participants (12 females and 16 males) with ages ranging from 18 to 49 years (*M* = 22.54), and for Experiment 3 the sample was 28 participants (12 females and 16 males) with ages ranging from 18 to 30 years (*M* = 21.93). All participants were native Japanese speakers.

#### Materials

The same materials as in Experiment 1 were used. In these experiments, size judgment tasks were included also in the recall phase (between each response). Additionally, at the end of the task, one transfer cycle (i.e., two filler lists and one Hebb list without any distractors) was added.

#### Procedure

The same task procedure as in Experiment 1 was followed, with 21 trials (seven cycles) instead of 24 (eight cycles) and a modification on the time given to respond to the size judgment task. Results of Experiment 1 showed that performance in both memory and size judgment task were very high, an average score of 0.80 and 0.88, respectively, and that the participants responded to the size judgment task in an average time of 0.96 s. Oberauer et al. (2015) developed an experiment in which they reduced the maximum time to respond to the size judgment task to 1.2 s (high cognitive load) and found that shortening the time affected memory and size judgment accuracy; however, it did not affect the presence and size of the Hebb repetition effect. Taking this information into account, in Experiment 2, we decided to modify the maximum time given for the size judgment task from 2 s to 1.2 s. As in Experiment 1, after the last letter is presented, a red question mark was shown, prompting the participants to recall the memory items in the same order as presented by pressing the letters on the keyboard. The entered letter was displayed for 0.3 s, followed by four size judgment tasks, then another red question mark appeared, and so on for the eight letters on the memory list. Finally, after the main task has finished, one transfer cycle was included, which proceeded in the same way as the previous trials, except it did not include any size judgment task. In Experiment 3, to reduce the difficulty of the task, the number of size judgment tasks between each memory item was reduced from four to two, and the maximum time to respond was increased back to 2 s.

### Results

We followed the same data analysis as in Experiment 1, adding the transfer trial analysis, in which we tested the hypothesis that the cumulative learning from the Hebb repetition effect in the complex span task can be transferred to a simple span task.

#### Memory accuracy

**Fig. 3:**
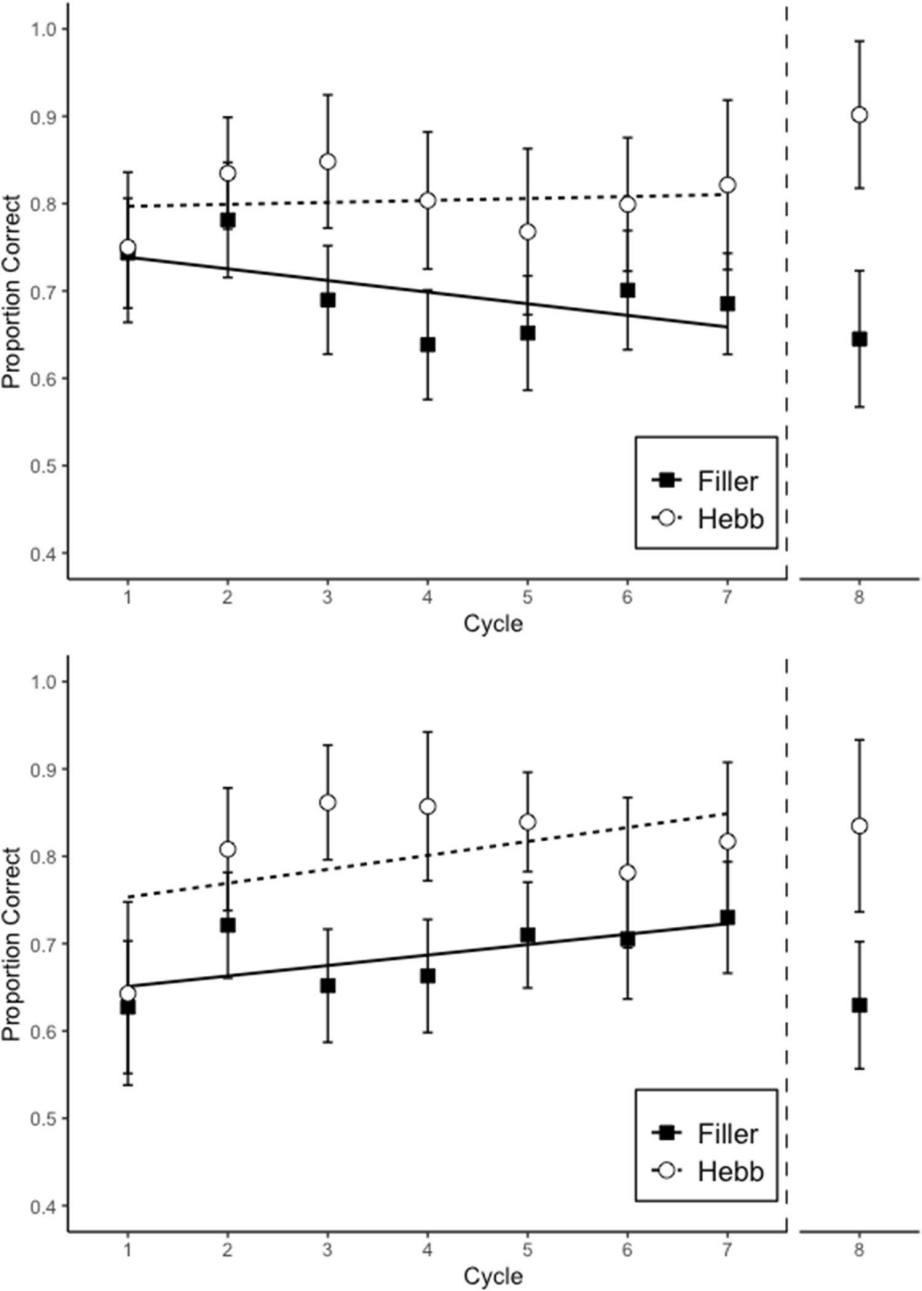
Memory accuracy in Experiment 2 (top) and Experiment 3 (bottom). Error bars are 95% CIs for within-subject comparisons (Bakeman & McArthur, 1996). The CIs can be interpreted in terms of classical null-hypothesis tests for pair-wise comparisons between data points: Two means differ significantly (p < .05) when their CIs overlap by less than 50% of the interval between each mean and the corresponding CI boundary (Cumming & Finch, 2005). The straight lines are regression lines estimated from fitting a linear model (Cycles 1 to 7); cycle 8 corresponds to the transfer trial

Memory performance was scored as the proportion of letters recalled in their correct within-list position. Figure 3 shows proportion of correct answers by cycle and repetition (Filler vs. Hebb). Table 1 summarizes the BFs reflecting the strength of evidence for the main effects and the interaction. There was no evidence for an interaction (Experiment 2)—rather, there was strong evidence for the additive model, which included cycle and repetition, but not the interaction (Experiment 3). The main effect of cycle was nonexistent in Experiment 2 and substantial in Experiment 3. There was strong evidence for the main effect of repetition in both experiments, which reflects the Hebb repetition effect. In this case, the mean effect size was 0.11, with a 95% credible interval of 0.06–0.15. Based on these results, we can say that the Hebb repetition effect increases memory performance in complex span by 6–15 percentage points over seven list repetitions.

#### Size judgment performance

**Fig. 4:**
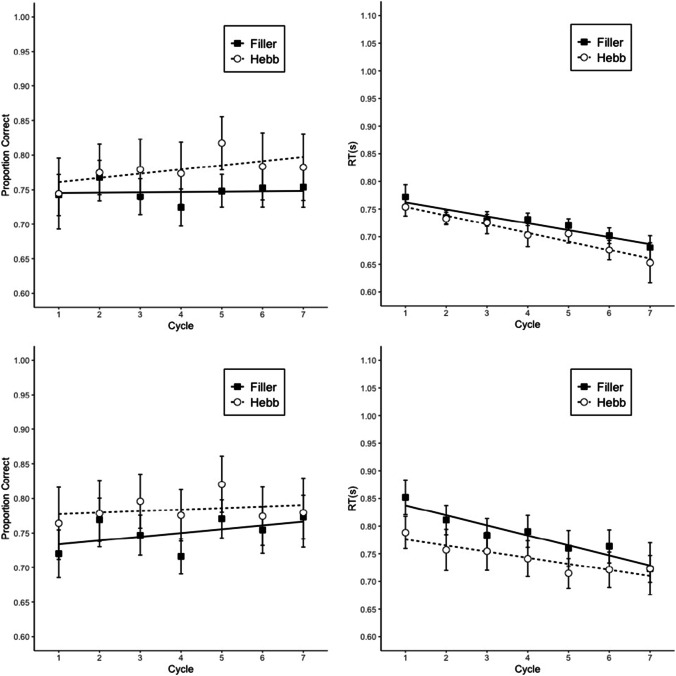
Performance in the size-judgment task in the encoding phase (top) and recall phase (bottom) in Experiment 2. Error bars are 95% CIs for within-subject comparisons. The straight lines are regression lines estimated from fitting a linear model

**Fig. 5:**
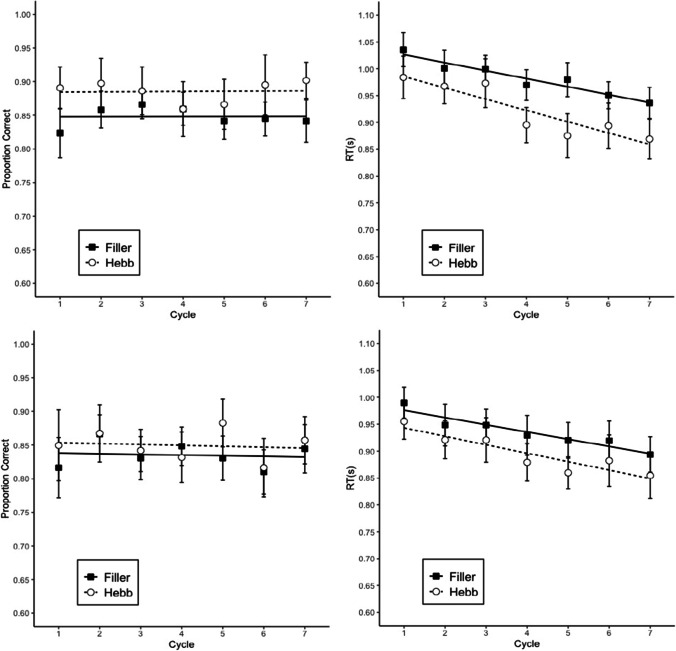
Performance in the size judgment task in the encoding phase (top) and recall phase (bottom) in Experiment 3. *Error bars are 95% CIs for within-subject comparisons. The straight lines are regression lines estimated from fitting a linear model*

The BFs are shown in Table 1, and the proportion of correct answers and RTs are presented in Figs. 4 and 5. The data were analyzed separately for the distractors in the encoding and recall phases. Regarding accuracy, there was no evidence for the interaction and no evidence for the main effect of cycle on either phase; a main effect of repetition was found on both phases in Experiment 2 and only on the encoding phase in Experiment 3. As for the RTs, there was no evidence for the interaction, and strong evidence for the main effect of both repetition and cycle in both phases and both experiments. As in memory accuracy, the list repetition had a beneficial effect on both accuracies and RTs.

#### Transfer trials performance

Memory accuracy in the transfer trials was scored as the proportion of letters recalled in their correct within-list position. Cycle 8 of Fig. 3 shows the proportion of correct answers by repetition. A Bayesian paired-samples *t* test showed that the repeated list had a higher proportion of correct responses than the filler lists, BF 31.5 (Experiment 2) and BF 6.23 (Experiment 3).

### Discussion

Experiments 2 and 3 showed a strong effect of repetition, even with distractor tasks in both encoding and recall phases. However, there was no evidence of an interaction between cycle and repetition.

One limitation of Experiment 2 was that most participants had a size judgment accuracy percentage lower than 80% in both encoding and recall phases (18 out of 28 and 27 out of 28 participants, respectively). This means that the participants might not have been paying attention, and randomly responding to the size judgment task in order to make it easier to remember the memory items. That scenario would transform the main task from complex to simple span, undermining our objective of minimizing the possibility of the participants to form the integrated representations. This situation made it necessary to run a third experiment in which we could measure the Hebb repetition effect while participants demonstrably engaged with the distractor task. After lowering the cognitive load, Experiment 3 successfully replicated the results of Experiment 2, without the distractor task accuracy problem. Most of the participants had more than 80% of accuracy on the size judgment task in the encoding phase (22 out of 28) and more than 70% in the recall phase (22 out of 28).

The data from Experiment 3 demonstrated a strong Hebb repetition effect, confirming that this type of learning can occur regardless of the interruptions in the sequence. Additionally, the results from the transfer trials also showed that the accuracy of recall from the repeated list is significantly higher than the one in the nonrepeated lists. This confirms that the participants could successfully transfer learning from the list in the complex span task to a simple span task trial.

Why would this occur? One possibility to consider is that participants removed the distractors from working memory after having processed them. This would enable them to form a chunk that includes only the list items that still remain in working memory. However, completely removing the irrelevant information (i.e., distractors) from working memory requires to have free time after processing the distractors (Oberauer et al., 2012b; Oberauer & Lewandowsky, 2016). In Experiment 2, which imposed high cognitive load (i.e., 1.2 seconds to respond to each distractor task), we tried to minimize the opportunity for removing distractors. This should have made it difficult to form integrated chunk representations that include only the memory list, excluding distractors. The results from the transfer trial can give us some information on this issue, as being able to successfully transfer the learning of the sequence in the complex span task to a simple span task could mean that the exclusion of irrelevant information from the acquired long-term memory representation is possible. The results from both experiments showed substantial evidence for the Hebb repetition effect on a simple span trial after seven repetitions in the complex span task.

Finding the Hebb repetition effect in a complex span task where the distractors are in both encoding and recall phases gives us important evidence towards a different mechanism for Hebb repetition learning in a complex span task, because it was assumed that Hebb repetition learning cannot occur when items of the repeated list are not following each other immediately (Cumming et al., 2003; Hitch et al., 2005). However, the strong transfer effect suggests that the mechanism could be the same in simple and complex span tasks.

## Experiment 4

In the prior experiments the memory items (letters) and the stimuli for the distractor task (words referring to concrete objects) belonged to clearly distinct classes, which made them easily distinguishable. This could have enabled participants to keep the distractor representations separate from the representation of the memory list in working memory, or selectively remove the distractors from working memory entirely. In that way, they could construct a representation of the memory list uninterrupted by distractors, and gradually acquire a unified, chunked representation of that list that excludes distractors. In the present experiment, we increased the challenge for such a mechanism by making memory items and distractor stimuli less discriminable: Both list items and distractors were Latin letters.

Two further considerations motivated Experiment 4: First, Experiments 2 and 3 showed no interaction between cycle and repetition. As mentioned earlier, this can be explained by the fact that performance in the Hebb lists was much higher than the nonrepeated lists from very early in the task, causing a ceiling effect which led to a nonlinear relationship. With less distinctive distractors, Hebb repetition learning might proceed slower, if it occurs at all. Second, the Japanese participants might have been subjected to stronger proactive interference in the laboratory due to having less exposure to alphabetical letters in their everyday life. Therefore, we test English speaking participants in Experiment 4, expecting that the filler lists will show stable memory performance, rather than decreased performance over cycles.

### Method

#### Participants

Participants were 50 volunteers recruited via Prolific Academic (Prolific AC) who took part in a single 45-minute session in exchange for ﻿£9. Inclusion criteria was as follows: (1) native English speaker; (2) nationality must be from the UK, USA, Canada, Australia, or New Zealand; (3) approval rating of at least 90% on prior submissions at Prolific AC; (4) normal or corrected-to-normal vision; (5) no cognitive impairment or dementia; (6) age between 18 and 30 years. Fifteen participants had to be excluded from the analysis due to incomplete data. Consequently, the total sample was 35 participants (19 females and 16 males) with ages ranging from 18 to 30 years (*M* = 23.2).

#### Materials

The same materials as in Experiment 3 were used for the memory list. The distractor task was a rhyme judgment task; we used the letter list and procedure from Jarrold et al. (2010): Participants had to decide whether a pair of uppercase letters rhymed or not. The letter pairs were created from the following 12 letters: A, C, D, E, G, I, J, K, P, T, V, Y. The pairs were selected at random on every trial, including on the repetition trials, with the only condition that throughout the task 50% of the pairs rhymed and 50% did not rhyme. The transfer cycle was constructed in the same way as in Experiments 2 and 3.

#### Procedure

The same task procedure as in Experiment 3 was followed, except with 18 trials (six cycles) instead of 21 (seven cycles) and a rhyme judgment distractor task. The reason of the reduced number of cycles is to shorten the duration of the online experiment.

### Results

We followed the same data analysis as in Experiments 1–3.

#### Memory accuracy

**Fig. 6:**
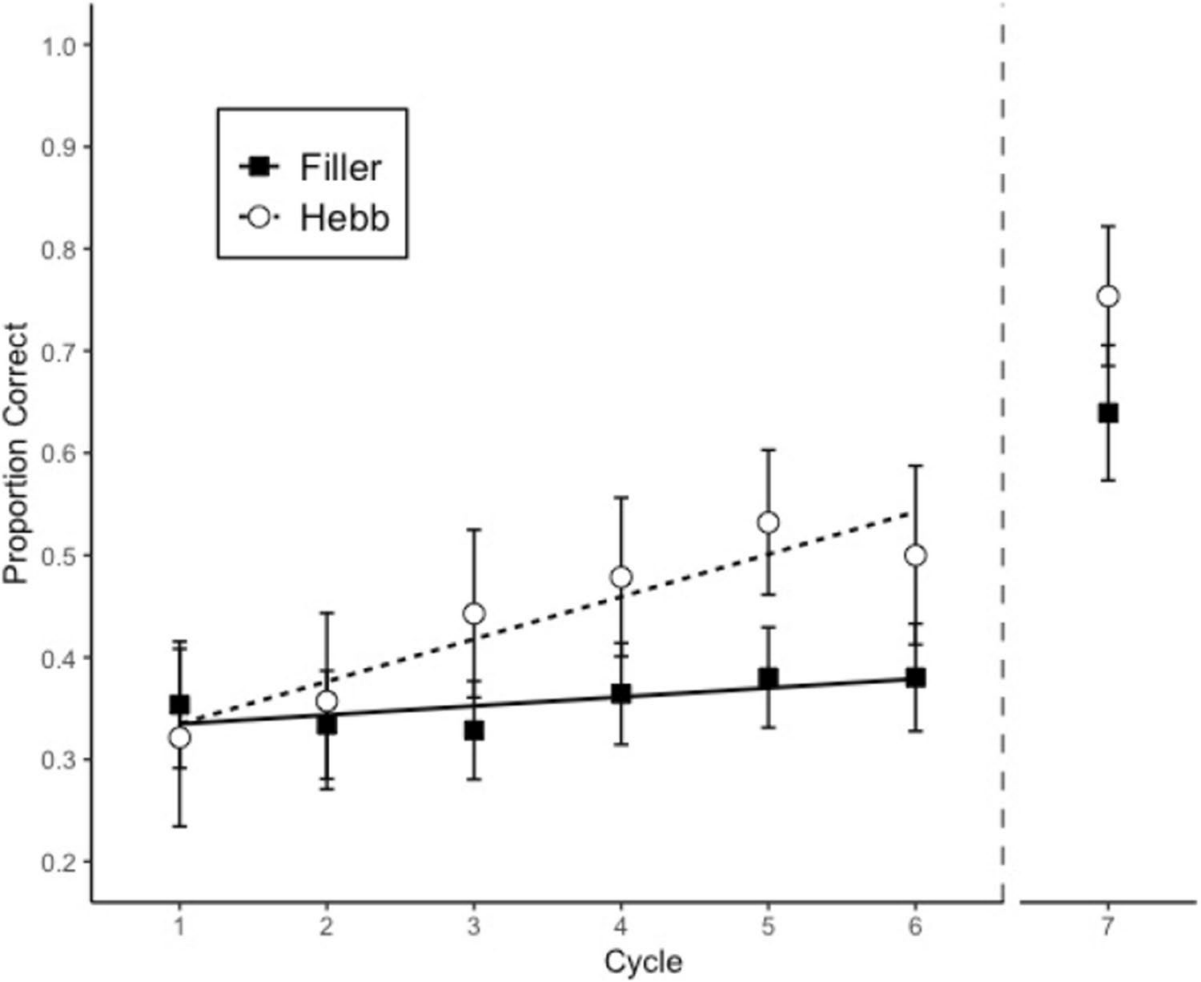
Memory accuracy in Experiment 4. Error bars are 95% CIs for within-subject comparisons (Bakeman & McArthur, 1996). The CIs can be interpreted in terms of classical null-hypothesis tests for pair-wise comparisons between data points: Two means differ significantly (p < .05) when their CIs overlap by less than 50% of the interval between each mean and the corresponding CI boundary (Cumming & Finch, 2005). The straight lines are regression lines estimated from fitting a linear model (Cycles 1 to 6); Cycle 7 corresponds to the transfer trial

Memory performance was scored as the proportion of letters recalled in their correct within-list position. Figure 6 shows proportion of correct answers by cycle and repetition (Filler vs. Hebb). Table 1 summarizes the BFs reflecting the strength of evidence for the main effects and the interaction. The analysis showed substantial evidence for the interaction, and strong evidence for the main effect of cycle and repetition. The mean effect size of repetition was 0.07 with a 95% credible interval of 0.04–0.11. Therefore, the Hebb repetition effect increased memory performance by 4–11 percentage points over six list repetitions.

#### Rhyme judgment performance

**Fig. 7:**
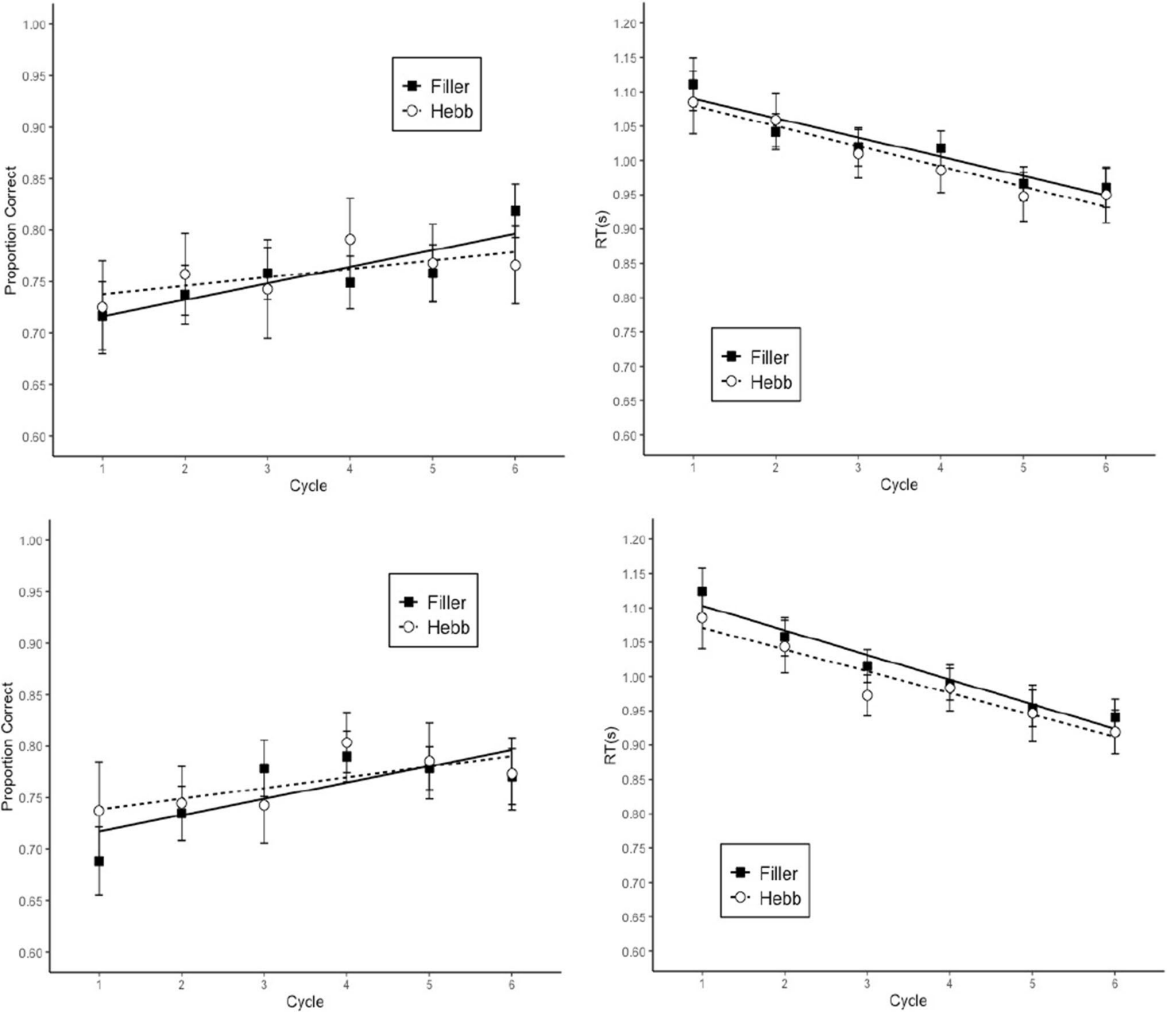
Performance in the rhyme judgment task in the encoding phase (top) and recall phase (bottom) in Experiment 4. Error bars are 95% CIs for within-subject comparisons. The straight lines are regression lines estimated from fitting a linear model

The BFs are shown in Table 1 and the proportion of correct answers and RTs are presented in Fig. 7. The data were analyzed separately for the distractors in the encoding and the recall phase. However, both phases showed the same results; no evidence for the interaction, nor for the main effect of repetition, for accuracy and RTs, and a strong main effect of cycle overall. The list repetition did not have a beneficial effect on the distractor task.

#### Transfer trials performance

Memory accuracy in the transfer trials was scored as the proportion of letters recalled in their correct within-list position. Cycle 7 of Fig. 7 shows the proportion of correct answers by repetition. A Bayesian paired-samples *t* test showed that the repeated list had a higher proportion of correct responses than the nonrepeated lists, BF 64.0.

### Discussion

Experiment 4 addressed the concern that in the preceding experiments the memory items and the distractors were highly distinctive. The first three experiments employed concrete Japanese words for distractors and alphabet letters for memory items, facilitating the distinction between distractors and memory items. The distinctiveness between these elements could reduce interference effects and make it easier to selectively remove distractors from working memory while maintaining the items. In that case, the distractors might not disrupt the formation of the integrated list representations that are thought to support the Hebb repetition effect (Cumming et al., 2003; Hitch et al., 2005). Therefore, it was necessary to maximize the challenge for a chunking process of learning.

The overall proportion of correct responses was markedly lower compared with the previous experiments, probably due to the reduced distinctiveness of distractors and items. Despite that difficulty, the results showed a clear Hebb repetition effect, as the Hebb list significantly increased in accuracy across cycles, whereas the Filler lists stayed constant. The presence of the Hebb repetition effect under these conditions corroborates the results of Experiments 2 and 3. We also replicated the transfer of learning from the complex span to the simple span task.

## General discussion

The objective of the present study was to confirm that Hebb repetition learning is possible in a complex span task, as it is in a simple span task. Oberauer et al. (2015) found the first evidence of this type of learning in a complex span task, and a series of experiments in the current study confirms that finding. Experiment 1 successfully replicated the results found by Oberauer et al. (2015). Experiments 2 and 3 showed that the Hebb repetition effect is still present even when distractors were included in between memory items while encoding *and* recalling them. Experiment 4 confirmed the Hebb repetition effect in complex span tasks, even when the to-be-remembered items and the distractors were similar in class.

Results from previous studies that tested partial Hebb repetition learning (Cumming et al., 2003; Hitch et al., 2005) have led to the question of whether a Hebb repetition effect will be observed in complex span (Oberauer et al., 2015). Those studies have established that not only a constant relation between items and their list positions is relevant, but also the constancy of the relation of items to neighboring items is necessary for the Hebb repetition effect to occur. However, with the evidence found by Oberauer et al. (2015) and the results from the present study we can confirm that Hebb repetition learning is possible in complex span tasks, in which the repeated sequence of items was disrupted by the intervening distractors.

We initially hypothesized that if the Hebb repetition effect could be found in a complex span task with distractors throughout the whole task, then the learning mechanism might be different from the one in simple span tasks. To test this hypothesis, we included a transfer cycle with two random trials and the repeated trial without any distractors (i.e., simple span) at the end of Experiments 2, 3, and 4. A strong transfer effect was found, which provides the first evidence of Hebb repetition learning being able to transfer from a complex span to a simple span task. Even though there was only one transfer cycle and one could expect it to not be powerful enough to show any effect, the results clearly indicate that the transfer effect exists in three experiments. Therefore, it is unlikely that the Hebb repetition effect in complex span and in simple span reflect completely different forms of learning that acquire different forms of knowledge.

Two different explanations can be given for the present findings: One is that the participants are able to successfully remove the irrelevant information (i.e., distractors) from working memory. This would enable them to create chunks of only the memory items. The other explanation is that they are learning position–item associations in long-term memory. In complex span, the distractors are not bound to position representations of their own, but are bound to the position of the preceding or the following item (Oberauer et al., 2012a, b; Oberauer & Lewandowsky, 2016). Therefore, in a complex span task, the first item is bound to Position 1, the second item to Position 2, and so on, and these bindings could be gradually learned in long-term memory when they are consistently repeated in the Hebb lists. Because the same position–item relations are repeated in the complex span Hebb lists, the learned position–item associations can be transferred to the simple span task.

Removal of nonnecessary information requires time (Oberauer et al., 2012b), and therefore it initially seemed unlikely that participants can completely remove multiple distractors while encoding and recalling, progressively creating chunks of memory items and generating a unified uninterrupted sequence. However, this possibility cannot be clearly dismissed, because distractors are encoded into working memory with reduced strength, about half of that of the memory items (Oberauer & Lewandowsky, 2019). If the distractors have a weaker representation in working memory, participants could be able to remove them and still form chunks consisting of only the memory items. As another way of creating a chunk for the memory list, the Hebb repetition effect could also be attributed to the creation of two separate streams (i.e., one for the memory list and one for the distractors; Farley et al., 2007; D. Jones et al., 1999). When processes of seriation are in play and the two stimuli are distinct enough, the participants could be able to create two separate lists (D. Jones et al., 1999; D. M. Jones & Macken, 1995) simultaneously. Experiment 4 reduced to a minimum those possibilities by drawing memory items and distractors, for both repeated and nonrepeated lists, from the same limited pool of stimuli. Previous research of the Hebb repetition effect in simple span tasks has shown that sampling successive lists from the same small pool of stimuli makes Hebb repetition learning more difficult than when lists are sampled from a larger pool (Page et al., 2013; Smalle et al., 2016; cf. St-Louis et al., 2019). In accordance with those results, our data shows that using the same small set of stimuli and making the items less discriminable has a negative effect on memory accuracy. However, it does not influence the Hebb repetition effect.

We considered the position–item association theory as well, which by itself did not seem to explain the Hebb repetition effect in simple span tasks (Burgess & Hitch, 2006;Cumming et al., 2003 ; Hitch et al., 2005), as both position–item relations and relations between neighboring items need to be constant for the occurrence of the effect. Recent studies, however, have reported findings that indicate the possibility of long-term learning of repeated position–item associations. Nakayama and Saito (2017) conducted a series of experiments in which they manipulated the positional frequency of nonwords, presenting some items more frequently at the same serial position in verbal sequences than others, and demonstrated gradual position–item learning in a Hebb-like repetition task. Moreover, Majerus and Oberauer (2019) in a series of serial recall tasks using words as memory items, showed that immediate serial recall improved as the same words were consistently presented in the same positions, even though the word-to-word transitions were not repeated. People learned individual position–item associations that were repeated across trials, which demonstrates that position–item association generates learning effects. Therefore, learning of position–item associations is a plausible mechanism also for the Hebb repetition effect in complex span tasks. As the present experiments have reduced the credibility of alternative mechanisms—in particular learning of item–item associations, and the formation of chunks—we argue that position–item association learning should be considered a viable hypothesis for explaining Hebb repetition learning in complex span, at least in a situation—as in the current experimental setting—where the repetition of a list selectively increases the frequencies of its item–position associations.

Based on these considerations, we tentatively hypothesize that both chunking and position–item association are suitable forms of long-term learning that can underly the Hebb repetition effect, and participants can choose to use one or the other depending on which one is more useful. In immediate serial recall studies, an uninterrupted repeated sequence seems to be the key for Hebb repetition learning (Cumming et al., 2003; Hitch et al., 2005), suggesting that chunking is the dominant learning mechanism. For example, in Cumming et al. (2003) participants were presented with a whole repeated list, and in that case, chunking is a good-enough mechanism to generate long-term learning. Position–item associations might not have been created in long-term memory simply because learning them was not necessary to improve performance. When participants needed to transfer their knowledge to a partially repeated list, the chunks that were previously created were no longer helpful, and therefore transfer was unsuccessful in the Cumming et al. (2003) experiment.

In our Experiments, if the participants used the chunking strategy while somehow excluding distractors from the chunks, then that knowledge was probably easily transferred to the final list without distractors. In contrast, if they were not able to use the chunking strategy, and instead utilized position–item associations to learn the list, then that knowledge was still useful for them to recall the repeated sequence when a simple span-like list was presented, as that list was consistent with the learned position–item associations. One prediction from this account is that a Hebb list learned in a simple-span task—when chunking is sufficient to learn it—cannot be transferred to the same list in a complex-span task because the list representation would not match. Thus, we expect the transfer effect between simple and complex span to be asymmetric.

To conclude, adding to the evidence by Oberauer et al. (2015), we were able to confirm that long-term learning of repeated memory lists occurs even with interrupted sequences. Furthermore, we obtained the first evidence showing that learning in a complex span task can be transferred to a simple span task. Until now, Hebb repetition learning has helped us to understand learning of words, which are uninterrupted phoneme sequences. The present study opens up further research avenues that can lead us to better understanding working memory and its relationship with much broader aspects of learning, namely, learning that is not restricted to successive sequences of elements.

## Footnotes

1 Oberauer et al. (2015) evaluated the main effects by estimating a null model with only the intercept and the random effect of subjects and comparing it to the cycle and repetition model. We conducted the same analysis with our data and found results that are consistent with the ones reported here.
